# Estimating the upper limit of prehistoric peak ground acceleration using an in situ, intact and vulnerable stalagmite from Plavecká priepast cave (Detrekői-zsomboly), Little Carpathians, Slovakia—first results

**DOI:** 10.1007/s10950-017-9655-3

**Published:** 2017-03-25

**Authors:** K. Gribovszki, K. Kovács, P. Mónus, G. Bokelmann, P. Konecny, M. Lednická, G. Moseley, C. Spötl, R.L. Edwards, M. Bednárik, L. Brimich, L. Tóth

**Affiliations:** 1 Department of Meteorology and Geophysics, University of Vienna, 1090 Vienna, Austria; 2 Geodetic and Geophysical Institute, Research Centre for Astronomy and Earth Science, Hungarian Academy of Sciences, Sopron, Hungary; 3 Institute of Geonics, Academy of Sciences of the Czech Republic, Ostrava, Czech Republic; 4 Planetarium Ostrava, Faculty of Mining and Geology, VSB-Technical University of Ostrava, Ostrava, Czech Republic; 5 Department of Earth Sciences, University of Minnesota, Minneapolis, USA; 6 Institute of Geology, University of Innsbruck, Innsbruck, Austria; 7 Geophysical Institute, Slovak Academy of Sciences, Bratislava, Slovakia

**Keywords:** Speleology, Stalagmite, Cantilever beam, Natural frequency, Peak ground acceleration, Prehistoric earthquake, Seismic hazard, Speleoseismology

## Abstract

Earthquakes hit urban centres in Europe infrequently, but occasionally with disastrous effects. Obtaining an unbiased view of seismic hazard (and risk) is therefore very important. In principle, the best way to test probabilistic seismic hazard assessments (PSHAs) is to compare them with observations that are entirely independent of the procedure used to produce PSHA models. Arguably, the most valuable information in this context should be information on long-term hazard, namely maximum intensities (or magnitudes) occurring over time intervals that are at least as long as a seismic cycle. The new observations can provide information of maximum intensity (or magnitude) for long timescale as an input data for PSHA studies as well. Long-term information can be gained from intact stalagmites in natural caves. These formations survived all earthquakes that have occurred over thousands of years, depending on the age of the stalagmite. Their ‘survival’ requires that the horizontal ground acceleration (HGA) has never exceeded a certain critical value within that time period. Here, we present such a stalagmite-based case study from the Little Carpathians of Slovakia. A specially shaped, intact and vulnerable stalagmite in the Plavecká priepast cave was examined in 2013. This stalagmite is suitable for estimating the upper limit of horizontal peak ground acceleration generated by prehistoric earthquakes. The critical HGA values as a function of time going back into the past determined from the stalagmite that we investigated are presented. For example, at the time of Jókő event (1906), the critical HGA value cannot have been higher than 1 and 1.3 m/s^2^ at the time of the assumed Carnuntum event (∼340 AD), and 3000 years ago, it must have been lower than 1.7 m/s^2^. We claimed that the effect of Jókő earthquake (1906) on the location of the Plavecká priepast cave is consistent with the critical HGA value provided by the stalagmite we investigated.

The approach used in this study yields significant new constraints on the seismic hazard, as tectonic structures close to Plavecká priepast cave did not generate strong earthquakes in the last few thousand years. The results of this study are highly relevant given that the two capitals, Vienna and Bratislava, are located within 40 and 70 km of the cave, respectively.

## Introduction

Damaging earthquakes in Central Europe are infrequent, but do occur. This raises the important issue for society of how to react to this hazard: Potential damages are enormous, and infrastructure costs for addressing these hazards are huge as well. Obtaining unbiased expert knowledge of the seismic hazard (and risk) is therefore very important.

Evidence of moderate and large historic (1906, Jókő) and paleoseismic events exists in the vicinity of investigated cave site. These observations imply that we need a better understanding of possible co-seismic ground motions in the nearby densely populated areas of Vienna and Bratislava.

The seismic hazard of an area of interest is specified in terms of the horizontal ground acceleration (or spectral acceleration) which shall not be exceeded in a certain time period with a given probability. Estimating seismic hazard appropriately, especially for critical infrastructure, requires information on the largest earthquake that has occurred in the past.

Most large earthquakes occur at plate boundaries. However, in territories with low or moderate seismic activity, such as intraplate areas, the recurrence interval of large earthquakes, belonging to the same source zone, can be as long as 10,000 years (Scholz 1990). Therefore in territories with low or moderate seismic activity, information about the largest quakes is usually not available since earthquake catalogues do not cover a sufficiently long time period. They are mostly based on observational periods less than 1000–2000 years. The lack of knowledge about these largest earthquakes is therefore usually balanced by assumptions about earthquake statistics and/or fault geometry. Such assumptions are difficult to quantify, rendering hazard estimation arbitrary and thus questionable.

In places where the recurrence interval between large events is on the order of thousands of years, the key to improve seismic hazard estimation is to obtain more reliable and realistic data regarding the frequency and magnitude of earthquakes. There is a critical need for additional observations that constrain the occurrence of the largest earthquakes and/or the maximum seismic acceleration over timescales of thousands of years.

Fragile geologic features such as precariously balanced rocks at Yucca Mountain, Nevada, have been used to quantitatively estimate the ground acceleration required to topple such boulders (Brune and Whitney 2000; Anderson et al. 2011). In situ stalagmites in caves represent another innovative archive which offers the advantage that these features can be dated. The ‘survival’ of vulnerable stalagmites requires that horizontal ground accelerations have never exceeded a certain critical value over time periods of thousand years or more. Such speleoseismological studies have been performed in several caves using an in situ approach (Lacave et al. 2000, 2004; Becker et al. 2006; Szeidovitz et al. 2005, 2008a, 2008b; Paskaleva et al. 2008; Gribovszki et al. 2008, 2013a, 2013b; Shanov and Kostov 2015) complemented by laboratory studies (Cadorin et al. 2001; Paskaleva et al. 2006; Bednárik 2009).

**Fig. 1:**
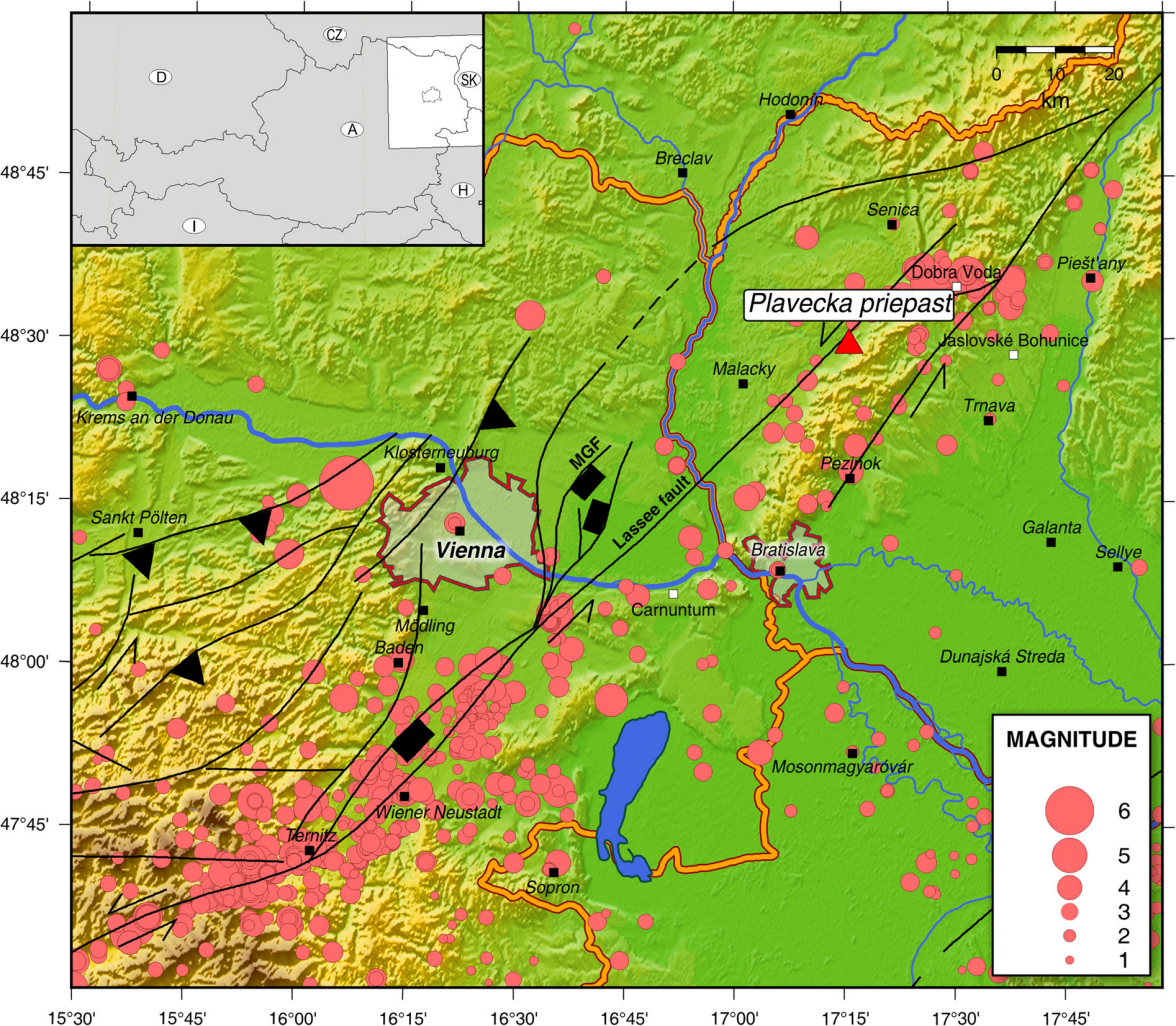
Location of the Plavecká priepast cave (red triangle) in the Little Carpathians of Slovakia, near the Vienna Basin Transfer Fault System (VBTF), two faults of which are the MGF (Markgrafneusiedler) and Lassee fault, as well as active faults (Horváth et al. 2004) and seismicity of the area (red circles) (Zsíros 2000; Tóth et al. 1996-2014). The *black lines* show the major faults of the area (thrust fault (*black triangle*), normal fault (*black rectangle*), strike slip fault (*black lines with arrow*)). The two amorphous areas are the two nearby capitals: Vienna and Bratislava. The general map, situated at the *top left hand side* of the figure, shows the position of the area of Fig. 1 in Austria and Slovakia. *Black squares* represent the large towns; *white squares* represent the location of objects that can be important at the point of view of seismic hazard

In the present study, we analysed a stalagmite in the Plavecká priepast cave, a dripstone cave in the Little Carpathians of western Slovakia, in the vicinity of the two capitals Vienna and Bratislava (Fig. 1). This intact and vulnerable stalagmite (IVSTM) is well suited for speleoseismological investigations, because of its candlestick shape and because it is sensitive enough (it has a sufficiently large height–diameter ratio (*H*/*D*)) to detect large paleoearthquakes. We compare our results to ground motion estimates from the 10 January 1906 Jókő earthquake.

## Scientific aim and applied method

The aim of this study was to estimate the upper limit for horizontal peak ground acceleration generated by earthquakes over historic and prehistoric epochs. One of the important factors regarding the seismic hazard of a given area is the degree of damage to buildings (earthquake proofness of buildings). From this viewpoint, the horizontal ground motion is more important than the vertical one since the buildings are more vulnerable for the horizontal ground motions than to vertical motions (Csák et al. 1981).

A specially shaped (candlestick shaped, i.e. high, slim and near-cylindrical form) IVSTM in the Plavecká priepast cave was chosen for the study. This IVSTM is suitable for this subject, because such IVSTMs survived all earthquakes that have occurred over their long ‘lifetime’—commonly thousands of years—depending on the age of the stalagmite. This requires that the horizontal ground acceleration has never exceeded a certain critical value during this time. Such information is very valuable, even if it concerns only a single geographic site.

Our investigation consists of the following steps: (i) in situ non-destructive determination of the natural frequency and the harmonic oscillations and measuring the dimensions of the IVSTM; (ii) laboratory measurements of the geomechanical and elastic properties (density, velocity of elastic waves propagating in stalagmite samples and tensile failure stress) of stalagmite samples; (iii) calculation of the natural frequency and the harmonic oscillations of the IVSTM and the static horizontal ground acceleration value (*a*_*g*_), which would break the IVSTM; (iv) age determination of core samples taken from speleothems; (v) determination of seismic wave attenuation with depth; and (vi) construction of a critical horizontal ground acceleration curve going back into the past.

Many reports can be found in the literature that interpret broken and tilted speleothems (soda straws, stalactites and stalagmites) as indicators of past earthquakes (Kagan et al. 2005﻿, ﻿Šebela 2008, Panno et al. 2009). For example, Forti (1997, 1998) and Lacave et al. (2004) presented a concise review about investigations of broken speleothems. One of the most questionable aspects of these studies is the fact that the reason for the fracture of the speleothems remains unclear. It might have been caused by an earthquake, but it can also have been broken by other processes (cf. Becker et al. 2006). For that reason, broken dripstones were not used in this study for making the final conclusions. Final conclusions have been done only by the investigation of intact, very vulnerable candlestick-shaped stalagmites. (Broken stalagmites have been used in this study only for determining the geomechanical and elastic parameters of the stalagmites originated exactly from the cave we investigated.)

## Site description

The Plavecká priepast cave is situated in Plavecký kras, the karstic region adjacent to the western margin of the central Little Carpathians. The cave is situated inside the hill on which the Plavecká Castle (Detrekő Vára) was built in the thirteenth century. The entrance of the cave is on the western slope of the castle hill near the Plavecké Pohradie village (Detrekőváralja). The cave was formed in Triassic limestones which contain layers of dolomite and belong to the Havranica nappe (part of Choč nappe—Mahel’ 1972).

**Fig. 2:**
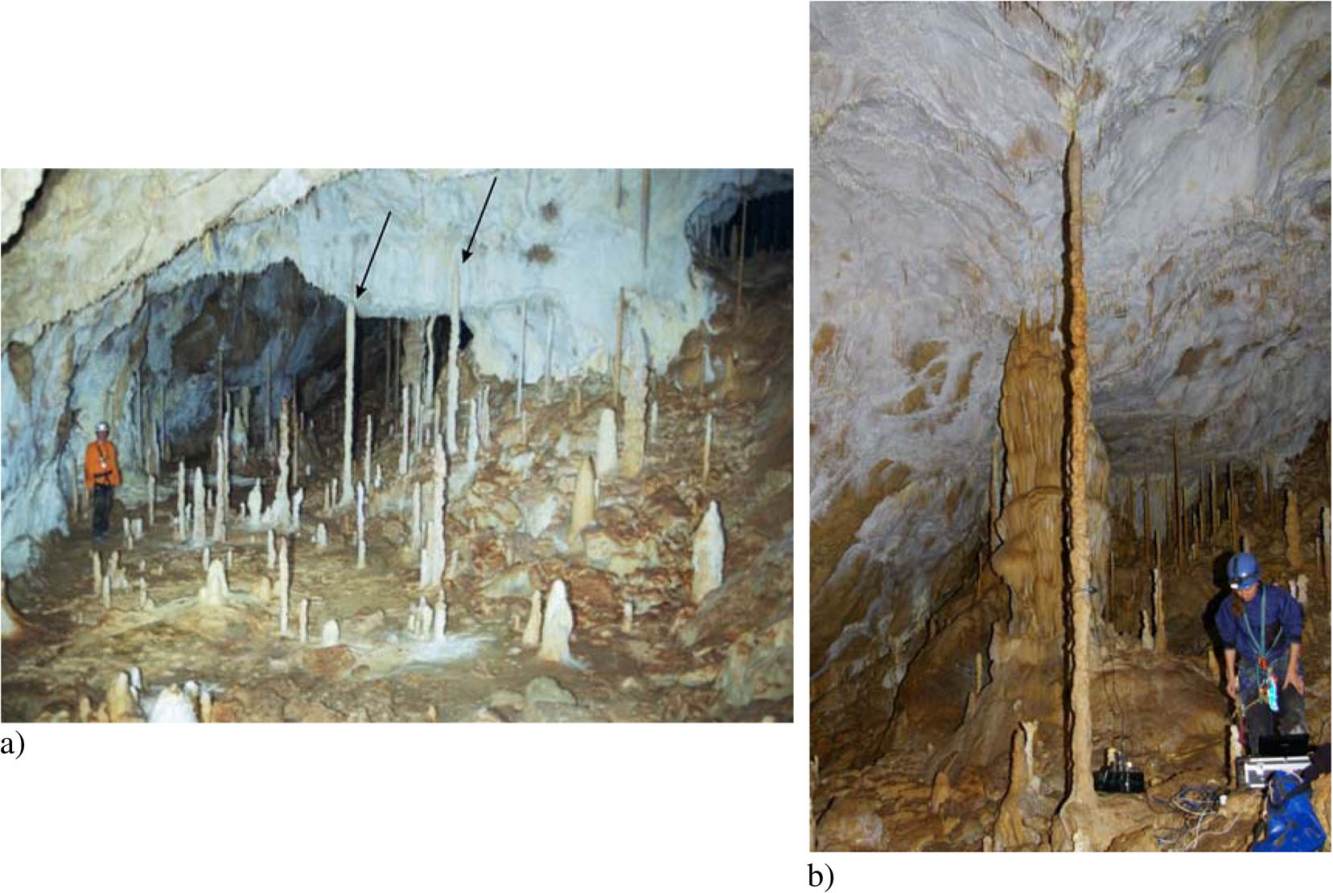
**a** Part of the Chamber of dripstones (Šmída 2010) in the Plavecká priepast cave. The *arrows* mark two stalagmites about 3 m tall. **b** Recording the vibration of the stalagmite we investigated in the rear of this chamber

**Fig. 3:**
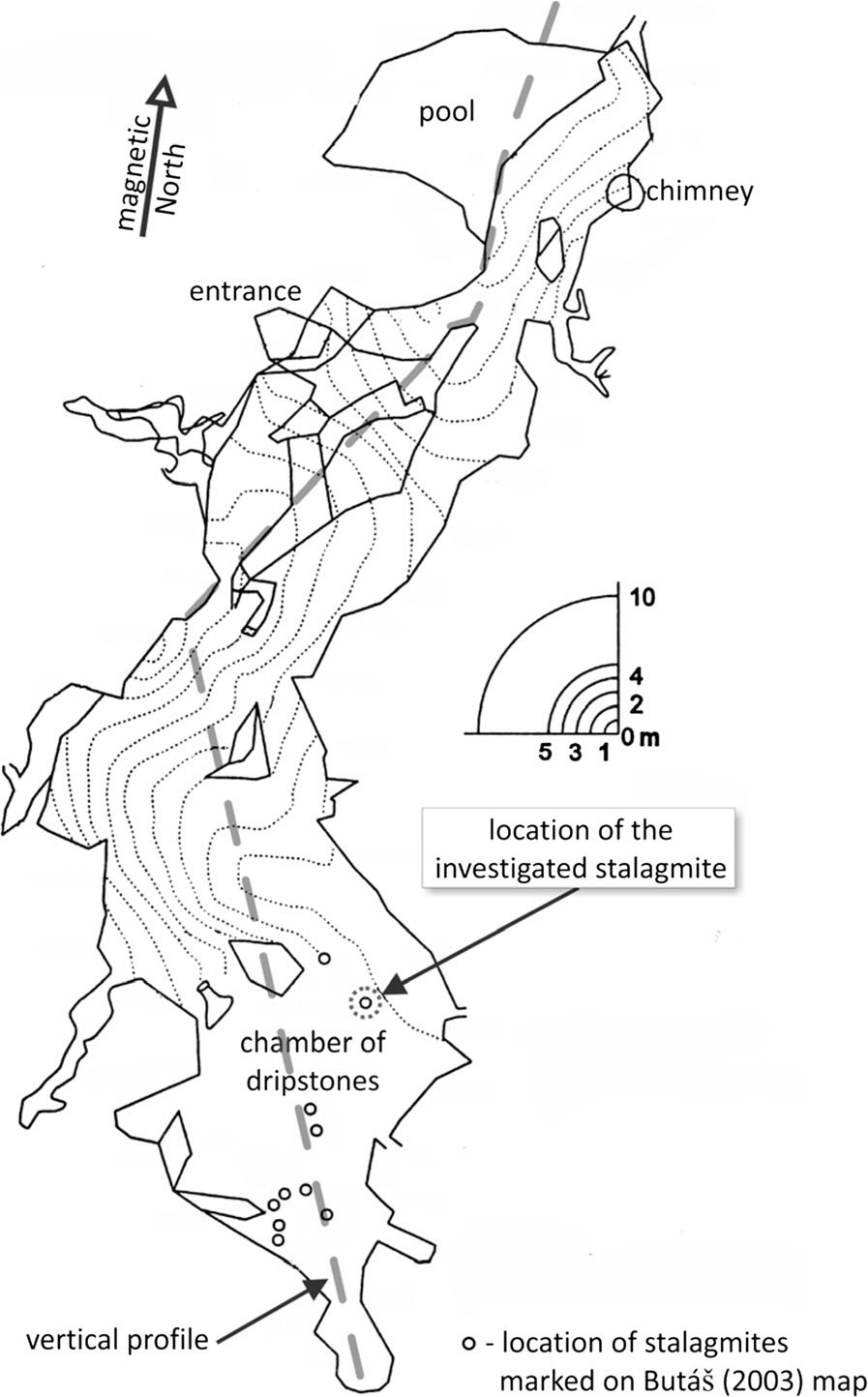
Plan view of the Plavecká priepast cave (after Butáš 2003). The *broken line* shows the trace of the vertical profile in Fig. 4

**Fig. 4:**
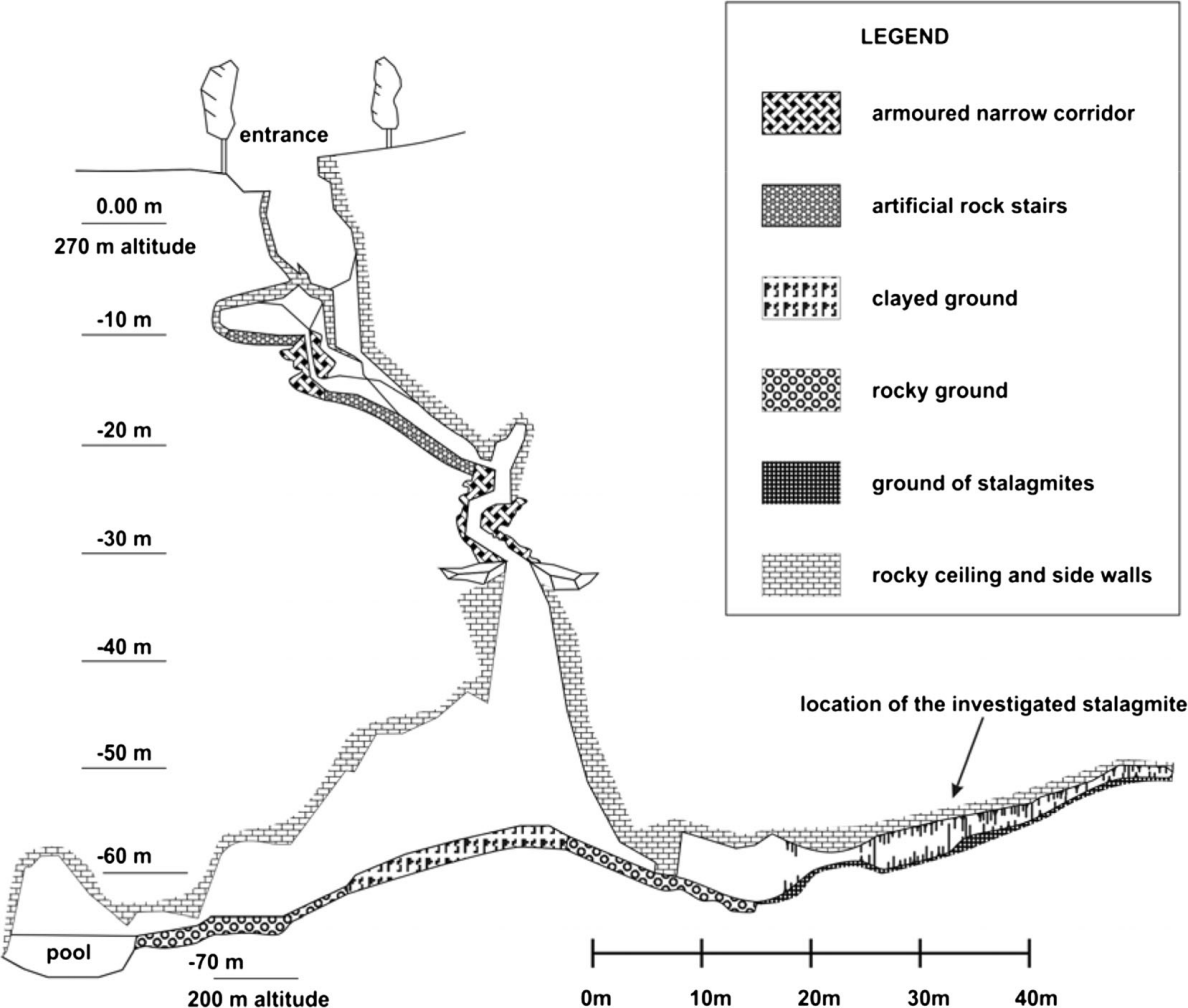
Vertical section of the Plavecká priepast cave (after Butáš 2003). The legend of the figure has been prepared by the authors

The cave is not open to the public but can be accessed via a 30-m-deep pit (Fig. 4). The stalagmite we investigated is located in the Chamber of dripstones of the cave (Figs. 2 and 3), which is the southern chamber of the cave. This chamber contains about 400 candlestick-shaped stalagmites. Their average height is about 1–2 m, and their average diameter is about 10 cm. Three stalagmites are considerably taller: Two are about 3 m (Fig. 2a), and the third is 4.3 m (Fig. 2b). We investigated the latter speleothem (Fig. 3).

**Fig. 5:**
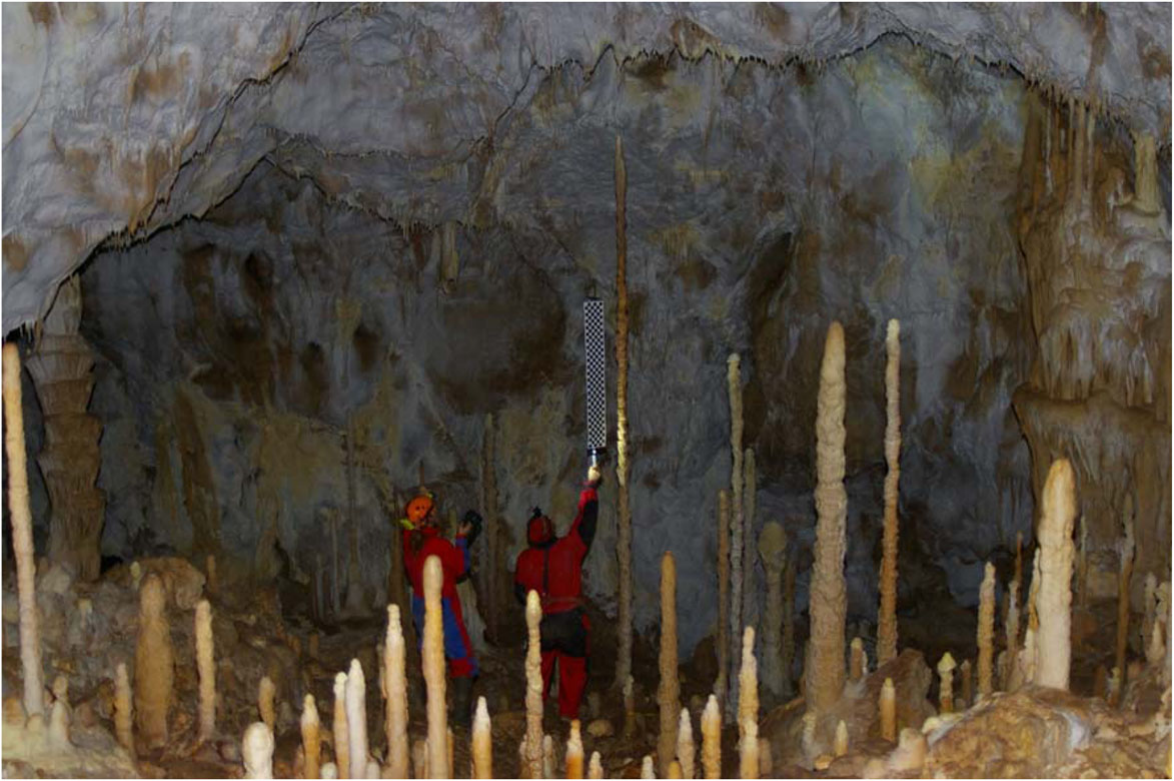
Measuring the height of the stalagmite we investigated. The length of the scale is 1.1 m

The total length of the cave is 335 m, and the width is 15–20 m. Its vertical extension is 70 m. More details about the cave and the speleothem formations can be found in Butáš (2005) and Šmída (2010) (Figs. 2, 3, 4 and 5).

Our research focused on the effect of earthquakes on intact and vulnerable stalagmites; it is therefore worth to mention Šmída’s (2010) work in Plavecká priepast cave. He maintained that it is nearly impossible that stalagmites in the Plavecká priepast cave could have remained intact since the early Pleistocene, because the edge of the Little Carpathians is a neotectonically active region. However, intact and vulnerable stalagmites of unknown age are abundant in this cave. If a strong earthquake had hit the area, these vulnerable stalagmites would have been broken (probably all stalagmites taller than 1–1.5 m; Šmída 2010).

The surroundings of the Plavecká priepast cave show evidence of historic and paleoseismic events. A moderate earthquake (*M* = 5.7) occurred on January 10 1906, 00:05 (local time) at Jókő (Dobra Voda) (Réthly 1907). The epicentre was only 20 km to the northeast of the cave.

The Mur–Mürz–Vienna Basin–Zilina line is a seismotectonically active, sinistral strike–slip fault, which passes by the Little Carpathians only about 5 km from the cave. The northern segment of the Mur–Mürz–Vienna Basin–Zilina line fault is the Dobra Voda segment (Beidinger and Decker 2011).

The Vienna Basin is within 35 km of the cave. The south-eastern part of the basin touches the Mur–Mürz–Vienna Basin–Zilina line. There are several normal faults in the Vienna Basin which belong to the Vienna Basin Transfer Fault System (VBTF). Prehistoric earthquakes were assumed to have occurred along these normal faults including the Carnuntum event (Decker et al. 2006) and several other earthquakes at the Markgrafneusiedler and Lassee fault. Trenches for paleoseismological investigations were excavated crossing the Markgrafneusiedl and Lassee faults. It has been stated that earthquakes with magnitudes ranging from 6.3 to 7 occurred from time to time at the Markgrafneusiedl fault during the last ∼120 ka (Hintersberger and Decker 2013). At least three major slip events (earthquakes?) since ∼20 ka before the present were identified on the Lassee fault with estimated magnitudes of ∼7 (Hintersberger and Decker 2014). These observations imply that the seismic potential of the Lassee fault segment might be much higher than suggested by historical seismicity, and the apparently seismically locked Lassee segment might represent a seismic gap along the VBTF.

The large number of vulnerable but still intact stalagmites and the close proximity of the Plavecká priepast cave to major active faults in this region, close to Bratislava and Vienna, and to the Bohunice nuclear power plant (Labák et al. 1998) make it a highly relevant site for testing whether earthquake-induced ground shaking has indeed occurred.

This study addresses several research questions which arise from the unique setting of the cave in relation to neotectonically active faults and the vicinity of two European capitals: (a) What is the maximum credible earthquake or the expected PGA along the Mur–Mürz–Vienna Basin–Zilina line fault in the vicinity of the cave (at the Dobra Voda and Lassee segments of the fault) on different timescales? (b) Can we confirm the occurrence of a large earthquake (magnitude between 6.0 and 6.3 ± 1, Decker et al. 2006, Hammerl et al. 2014) in the middle of the fourth century AD at the Lassee fault and/or other large earthquakes with magnitudes ranging from 6.3 to 7 that are assumed to have occurred on the Markgrafneusiedler and Lassee faults during the last ∼120 ka (Hintersberger and Decker 2013, 2014)? (c) Can we confirm the assumed seismic gap at the Lassee segment of the VBTF? (d) Could the stalagmite we investigated in the Plavecká priepast cave survive the ground motion of the moderate size (*M* = 5.7) Jókő earthquake, and how can we use that to improve ground motion prediction equations (GMPEs)?

## The investigated stalagmite

We identified three stalagmites in the southern chamber of the cave (Chamber of dripstones) which are slim, candlestick-shaped, intact and vulnerable (Figs. 2 and 5), and we studied the tallest and most vulnerable one. The investigated IVSTM is located about 40–50 m from the end of the 30-m-deep pit of the cave.

About 500 stalagmites, with a similar candlestick shape, are present in this cave, but many of them already form columns (stalagnate), i.e. their peaks touch the ceiling of the chamber. Compared to stalagmites, these columns are very stable formations, more resistant to horizontal ground acceleration than the stalagmites.

The height of the studied IVSTM is 4.3 m, and its average diameter is about 8.5 cm. The diameter of the stalagmite along the vertical axis changes slightly from about 10.5 cm near the base to about 7.0 cm in the top part.

The fundamental requirement of the method we used is that at least the upper third of the stalagmites must be completely intact, since our final conclusion refers to 6000 years (ka) before the present (see Fig. 14 in Sect. 9). The stalagmite we investigated has a candlestick shape with a diameter-height ratio larger than 50 and no parts where the diameter decreases significantly. The largest bending moment is associated with the weight of the stalagmite, affecting at the bottom part of the stalagmite during ground acceleration. The inhomogeneous structure within the stalagmite can modify the height (the position) at which the break would occur along the vertical axis. With less than 10% cavities and a cylindrical shape, the break would have to occur in the bottom third or half part of it as well (Gribovszki et al. in prep.).

Earlier works have shown that stalagmites with a *H*/*D* ratio larger than 20 are suitable for such a study, since their eigenfrequency is low enough to fall into the frequency range of modest local earthquakes (<∼25 Hz) and large remote earthquakes (∼2–∼10 Hz) (Lacave et al. 2000, 2004). In this frequency range, resonance can occur, an effect which we do not take into account here. In that case, failure can occur at a lower acceleration than predicted by our (static) determination.

**Fig. 6:**
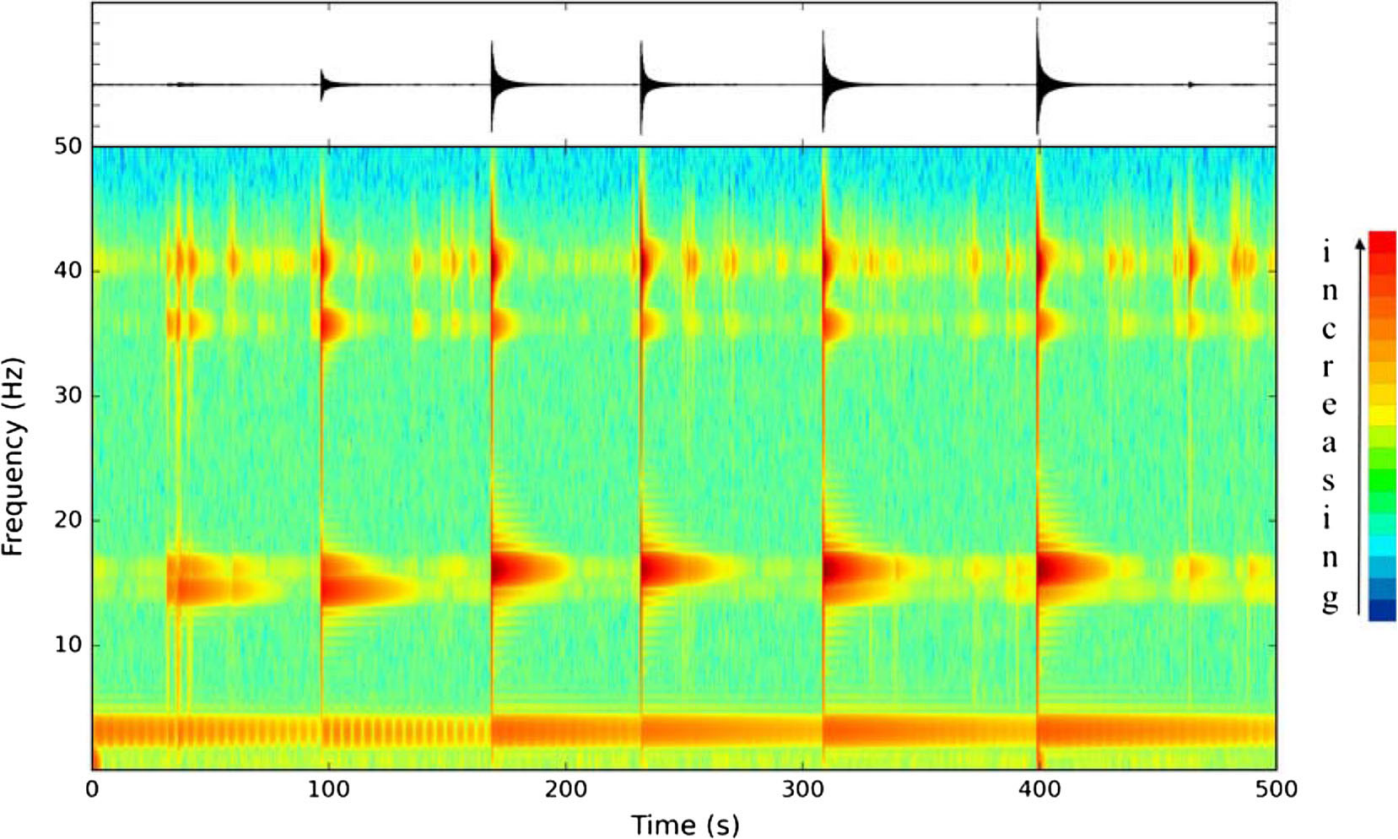
Vibration and power spectral density of the studied IVSTM along the recorded signal of the excited IVSTM. The *colour scale bar* that belongs to the spectrogram is at the *right side* of the figure

We measured the dimensions of the IVSTM (Fig. 5) and recorded its vibration (Figs. 2b and 6, Table 1) after gently knocking it. We did not record the vibration from ambient noise or microseismicity. Core samples for age determination were taken from this IVSTM as well (see details in Sect. 7).

**Table 1:** Dimensions and natural frequencies measured by non-destructive in situ examinations of the studied IVSTM

Height (m)	Diameter (cm)	*H*/*D*	Measured *f*_0_ (Hz)	Measured *f*_1_ (Hz)	Measured *f*_2_ (Hz)
4.30	Average 8.5 (10.5–7.0)	51	3	14.5, 16	36, 41

Our method is particularly applicable for stalagmites in shallow caves, since seismic waves attenuate with depth (Becker et al. 2006). The results are thus the stricter, the shallower the cave is. The IVSTM that we investigated was found in the Chamber of dripstones (Fig. 3) located at a depth at about 175 m below the highest point of the Plavecká Castle Hill, which is shallow enough for our method.

## Non-destructive in situ measurements

We only determined the IVSTM’s dimensions, natural frequency and harmonic oscillations, because in situ measurements had to be done non-destructively (all speleothems in Slovak caves are protected by law). In order to measure the natural frequency, a low-frequency geophone (type LF-24) was fastened onto the stalagmite. The geophone eigenfrequency is 1 Hz; it has a built-in non-linear correction preamplifier. A Reftek 130S-01 data logger was used (24 bits, AD converter). A special coupling sensor holder was built to ensure sufficient mechanical connection between the stalagmites and the geophone and to precisely adjust the horizontal position of the geophone.

The IVSTM was excited by a gentle hit with a finger. The vibration of the excited IVSTM was recorded (Fig. 6), and it was later analysed in the office. As shown in Fig. 6 and Table 1, the power spectral density of vibration indicates that the eigenfrequency of the IVSTM is around 3 Hz, and the first higher harmonics are about 14.5 and 16 Hz. Such a low natural frequency resonance likely occurs during a local earthquake (Lacave et al. 2004), since these values are below 20 Hz, which is within the frequency range of local earthquakes. Our theoretical calculations are based on the cantilever beam theory without resonance. This means that in reality, the IVSTM would break at lower horizontal ground acceleration values than the computed ones. In other words, the computed horizontal ground acceleration values provide an upper estimate of the maximum earthquake energy.

As shown in Fig. 6 and Table 1, the harmonic oscillations split into two parts, probably because the stalagmite we investigated was not completely axially symmetric (Budó 1968).

## Theoretical calculations and results of geomechanical and elastic laboratory tests

For a perfectly cylindrical stalagmite (i.e. constant diameter), simple equations can be deduced from cantilever beam theory (Cadorin et al. 2001; Lacave et al. 2000; Szeidovitz et al. 2008a). The natural frequency of a stalagmite would then be

1 $$ {f}_0\approx \frac{1}{\pi}\sqrt{\frac{3.1{ED}^2}{16\rho {H}^4}} $$

The horizontal ground acceleration resulting in a failure of a stalagmite would be expressed as

2 $$ {a}_g=\frac{D{\sigma}_u}{4\rho {H}^2} $$

where *D* is the diameter, *H* is the height of stalagmite, *ρ* is the mass density of the stalagmite, *E* is the dynamic Young’s modulus and *σ*_*u*_ is the tensile failure stress.

The harmonic oscillations of the cantilever beam calculated with the Bernoulli–Euler beam theory is (for a derivation, see, e.g. (41) in Kong et al. (2008))

3 $$ {f}_i=\frac{\omega_i}{2\pi}=\frac{{\left({s}_i L\right)}^2}{2\pi}\sqrt{\frac{EI}{\rho {AH}^4}} $$

where *I* is the second moment of cross section, *A* is the area of cross section and *ω*_*i*_ is the angular frequency.

*s*_*i*_ *L* = 1.875, 4.694, 7.855, 10.996, 14.137, *i* = (1, 2, 3, 4, 5).

For a cylindrical shape $I=\left(\frac{\pi}{64}\right){D}^4$, therefore Eq. (3) is modified (see, e.g. (45) in Kong et al. (2008)) as

4 $$ {f}_i={\alpha}_i\sqrt{\frac{ED^2}{16\rho {H}^4}} $$

where *α*_*i*_ = (*s*_*i*_ *L*)^2^ / 2π, and therefore, *α*_0_ = 0.559, *α*_1_ = 3.507, *α*_2_ = 9.820, *α*_3_ = 19.244 and *α*_4_ = 31.808.

**Fig. 7:**
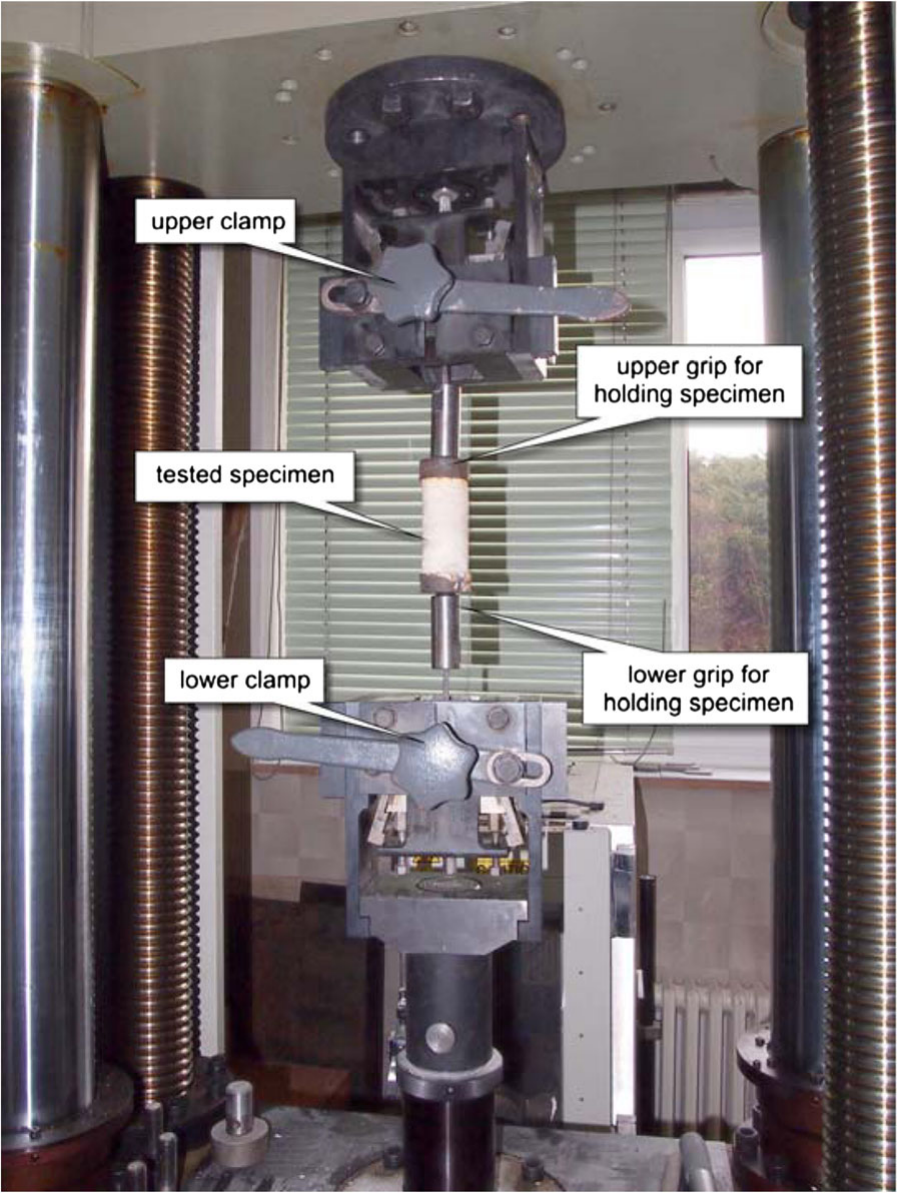
Pure tensile laboratory test on regular cylindrical-shaped stalagmite specimens from the Plavecká priepast cave. *Labels* refer to different parts of the apparatus used for the pure tensile laboratory tests

Values for density, ultrasonic Vp, Vs and tensile failure stress are based on laboratory measurements. Measurements were carried out in the mechanical laboratory of the Institute of Geonics of the Czech Academy of Sciences. Ultrasonic Vp and Vs velocities were measured by direct pulse transmission technique using digital ultrasonic portable instrument (Pundit Lab+, Proceq Company). The measurement was performed in the longitudinal direction of the stalagmite specimen, and the transducers we used had a frequency of 250 kHz. The dynamic Young’s modulus was calculated by using the results of ultrasonic Vp and Vs values (Nováková et al. 2011; Konecny et al. 2015). The tensile strength was determined by a pure tensile laboratory test on a mechanical press ZWICK 1494 equipped with grips to hold the specimen during the test (Fig. 7). The results are summarized in Table 2.

**Table 2:** Results of mechanical laboratory tests (average values)

	Density, *ρ* (kg/m^3^)	Ultrasonic, Vp (km/s)	Ultrasonic, Vs (km/s)	Dynamic Young’s modulus, *E* (MPa)	Tensile failure stress, *σ*_u_ (MPa)
Plavecká priepast	1940.5 ± 6.4	4.40 ± 0.18	2.10 ± 0.16	25,181.0 ± 3915.3	0.51 ± 0.13

Our results show that the average failure tensile stress values of broken stalagmite samples from the Plavecká priepast cave (*σ*_*u*_ = 0.51 MPa) are considerably lower than the values we measured from another cave system 230 km further east, e.g. from Olimposz hall in the Baradla cave (*σ*_*u*_ = 1.62 MPa) and Ördöglik hall in the Domica cave (*σ*_*u*_ = 2.75 MPa) which were determined by different, but comparable methods as Brazilian test (Szeidovitz et al. 2008a; Gribovszki et al. 2013b). On the other hand, the average failure tensile stress value in this study is very close to the minimum value (0.4 MPa) of failure tensile stress measured on broken stalagmites from Hotton cave (Belgium) (Cadorin et al. 2001) evaluated by a static bending test.

**Fig. 8:**
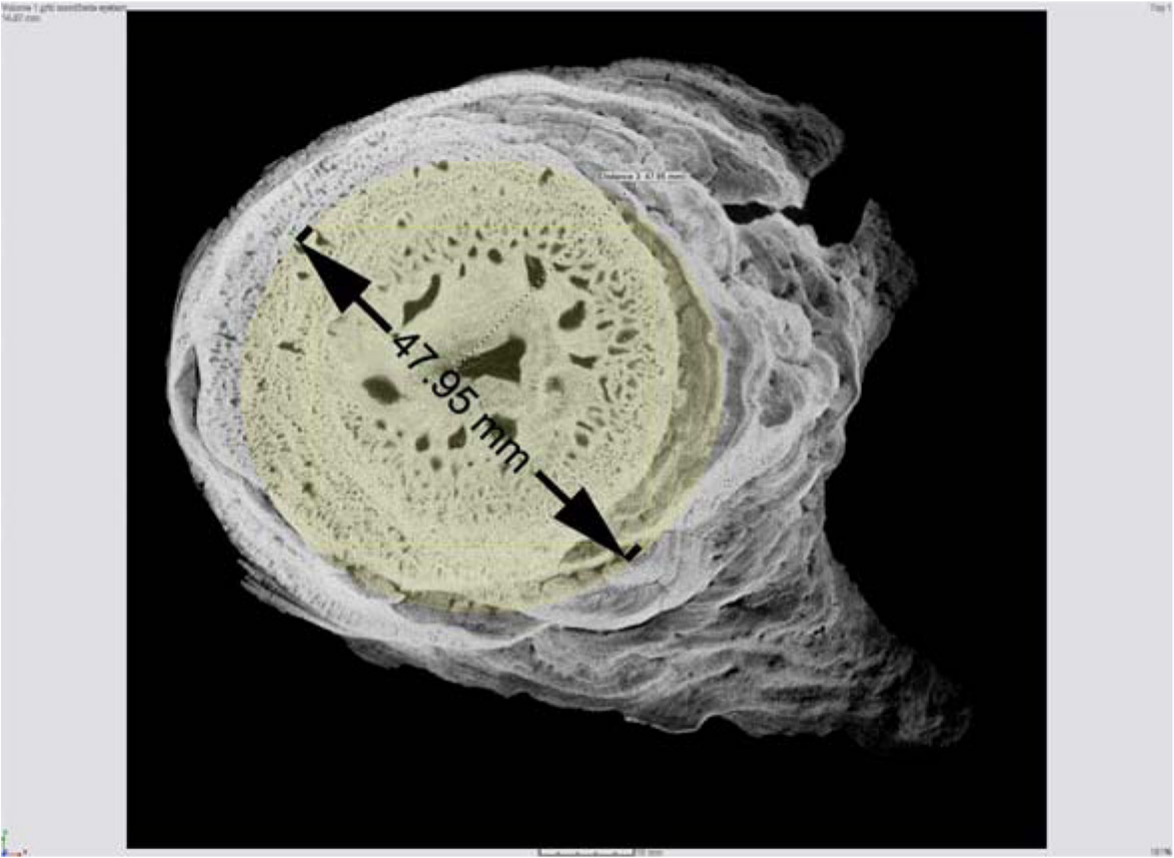
Tomographic image of a fallen stalagmite from the Plavecká priepast cave (by courtesy of Kamil Soucek). The region marked in *yellow* served to estimate physical dimensions

The tomographic image of a broken stalagmite from the Plavecká priepast cave (Fig. 8) helps to explain the low tensile failure stress values. This image demonstrates that the interior of the stalagmite is not homogeneous at all and reveals a relatively high proportion of air-filled pores. Any cavity corresponds to zero mechanical resistance, and the net tensile stress value must be smaller. The strength of the stalagmite is therefore less for dynamic effect as well.

The value of the dynamic Young’s modulus (25.2 GPa) is slightly higher than those from earlier caves we inspected, e.g. the Baradla cave (*E* = 20.8 GPa), the Domica cave (*E* = 23.6 GPa) and those from Hotton cave (*E* = 22.0 GPa) measured by Cadorin et al. (2001).

Table 3 shows the theoretical eigenfrequency (*f*_0_) and theoretical horizontal ground acceleration values that would result in failure (*a*_*g*_), based on Eqs. (1) and (2). The results of laboratory tests and the dimensions of the stalagmite were used for the theoretical calculations.

**Table 3:** Theoretical natural frequency and expected horizontal ground acceleration values for failure (see text)

Height (m)	Diameter (cm)	*H*/*D*	Measured *f*_0_ (Hz)	Theoretical *f*_0_ (Hz)	Theoretical *a*_*g*_ (m/s^2^)
4.30	Average 8.5	51	3	2.3	0.30

The theoretical eigenfrequency value (theoretical *f*_0_) is close to the measured value. The deviation may be a consequence of the approximations we made. Indeed, the real shape of the stalagmite was not truly cylindrical, and the dripstone substance was not homogeneous; the physical parameters used in the calculations were derived from mechanical tests of different stalagmite samples.

Horizontal ground acceleration resulting in a failure of the studied IVSTM is 0.30 m/s^2^ in the static case.

Tables 4 and 5 show in situ measured values compared with calculated values from Eq. (4), in two ways: (a) from the theoretical eigenfrequency and theoretical harmonic oscillation values (‘*theoretical-theoretical*’ approach) and (b) from the measured eigenfrequency and measured harmonic oscillation values (‘*theoretical-measured*’ approach).

**Table 4:** Measured and ‘*theoretical-theoretical*’ approach eigenfrequencies and harmonic oscillations (see text)

Height (m)	Diameter (cm)	*H*/*D*	*f*_0_ (Hz)		*f*_1_ (Hz)		*f*_2_ (Hz)	
			Theoretical	Measured	Theoretical-theoretical	Measured	Theoretical-theoretical	Measured
4.30	Average 8.5	51	2.3	3	14.4 = 2.3 × 6.274	14.5; 16	40.4 = 14.4 × 2.800	36; 41

**Table 5:** Measured eigenfrequency and harmonic oscillations and ‘*theoretical-measured*’ approach harmonic oscillations (see text)

Height (m)	Diameter (cm)	*H*/*D*	*f*_0_ (Hz)	*f*_1_ (Hz)		*f*_2_ (Hz)	
			Measured	Theoretical-measured	Measured	Theoretical-measured	Measured
4.30	Average 8.5	51	3	18.8 = 3 × 6.274	14.5, 16	40.6 = 14.5 × 2.80; 44.8 = 16 × 2.800	36, 41

The theoretical-theoretical harmonic oscillations *f*_1_ and *f*_2_ are almost the same as the measured *f*_1_ and *f*_2_ (in case of *f*_1_, the theoretical-theoretical value fits well into the measured lower *f*_1_ value, and in case of *f*_2_, the theoretical-theoretical value fits well to the measured higher *f*_2_ value), which means that using our calculations (Eq. (2)), the 8.5 cm as the average diameter of the stalagmite is adequate.

The theoretical-measured harmonic oscillations are higher than the measured ones (except *f*_2_ = 40.6), which indicates that the shape of our stalagmite is not completely cylindrical, but the diameter decreases upward along its axis. (Kegyes-Brassai Csaba personal communication).

## Age determination of the stalagmite

Speleothems are excellent archives of the paleoclimate and paleoenvironment (e.g. Fairchild and Baker 2012), because they grow over long periods of time in a relatively simple fashion: Stalagmites grow upward from the ground; therefore, younger segments are above the older ones. Chronological ages can be measured accurately and precisely using U-series techniques. Stalagmites, in particular, are considered as the best archives because of their regular internal growth structure.

It is still an open question whether earthquake-related deformation can also be obtained from stalagmites (e.g. Forti 2001; Becker et al. 2006) as the geometry of the stalagmite depends on the drip water (volume and speed of discharge, chemical composition, etc.) and the distance from the ceiling as well. Each layer of the stalagmite acts as a repository of its own growth history. Any change in the orientation or alignment of a stalagmite is likely to affect the growth pattern. These shifts may be due to ground deformation caused by an earthquake but could also be caused by other processes, e.g. a shift in the drip on the ceiling or subsidence of sedimentary ground on which the stalagmite rests, etc.

**Fig. 9:**
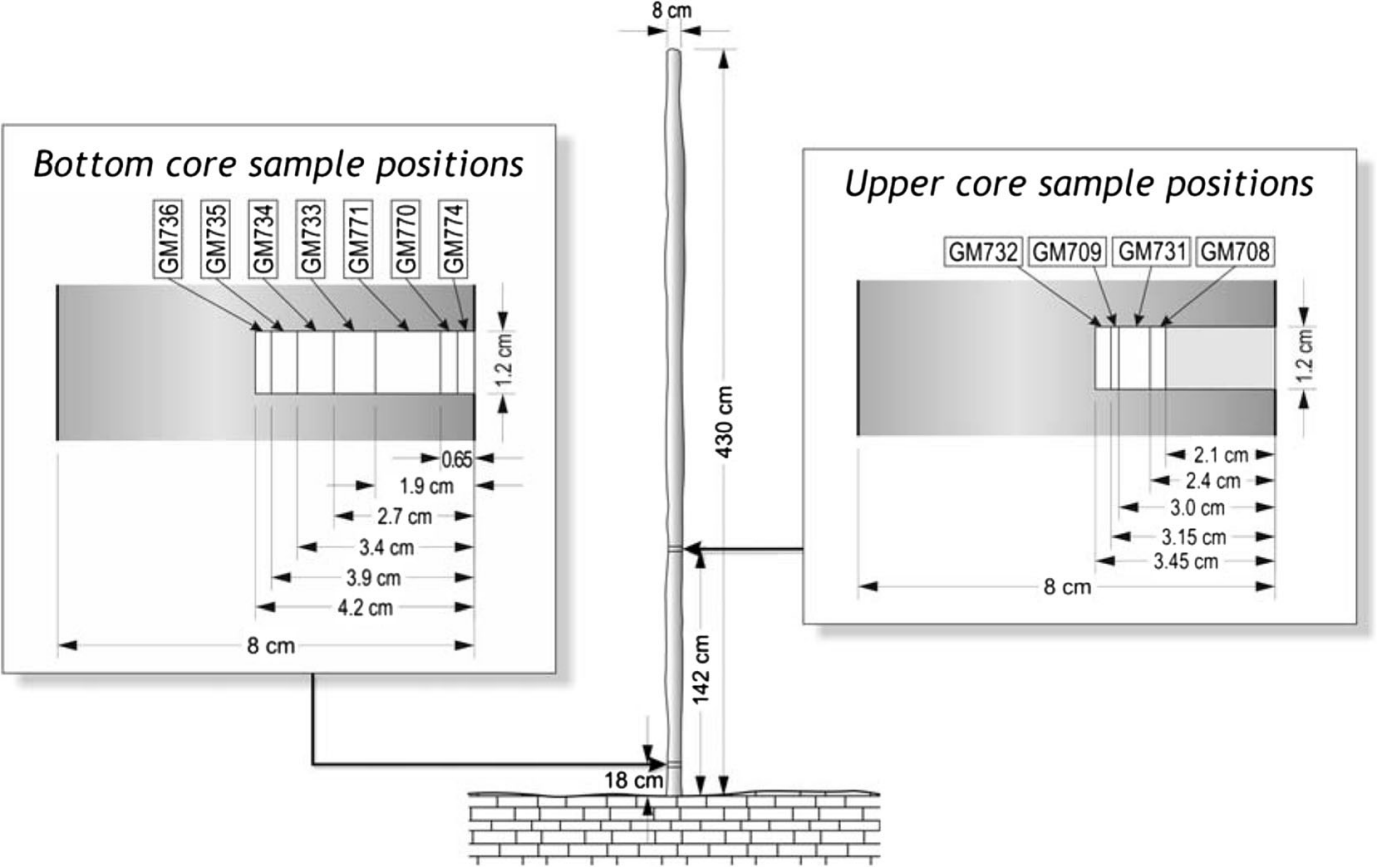
Location of the core samples drilled in IVSTM we investigated for age dating. ‘*Bottom*’ and ‘*Upper*’ refer to the positions of the cores at heights of 0.18 and 1.42 m

**Fig. 10:**
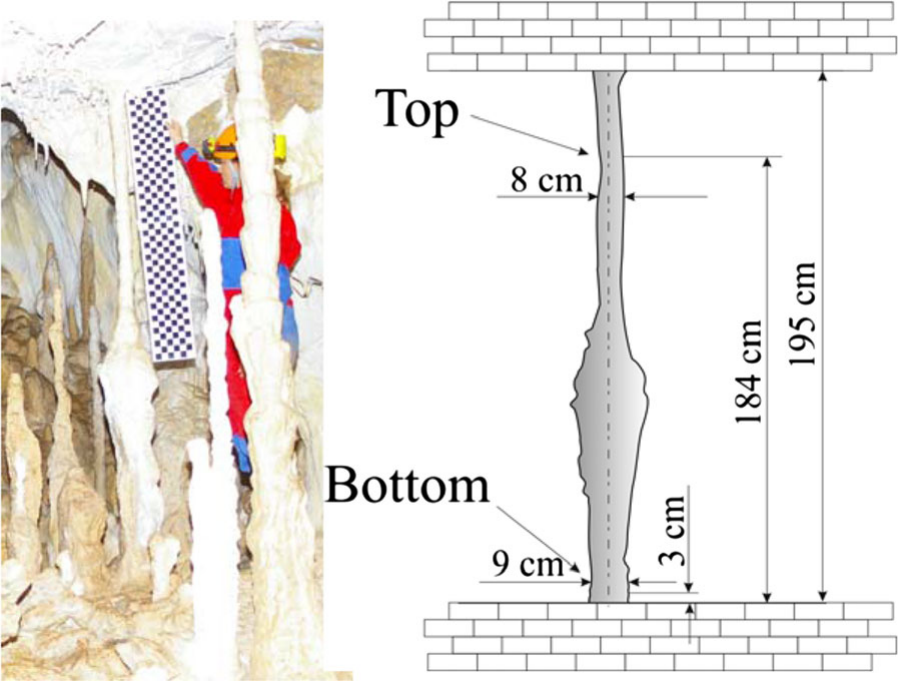
The 1.95-m in situ dripstone column used for age determination. ‘*Bottom*’ and ‘*Top*’ refer to the positions of the core samples, along the vertical axis of the dripstone, at heights of 0.03 and 1.84 m on the dripstone

**Fig. 11:**
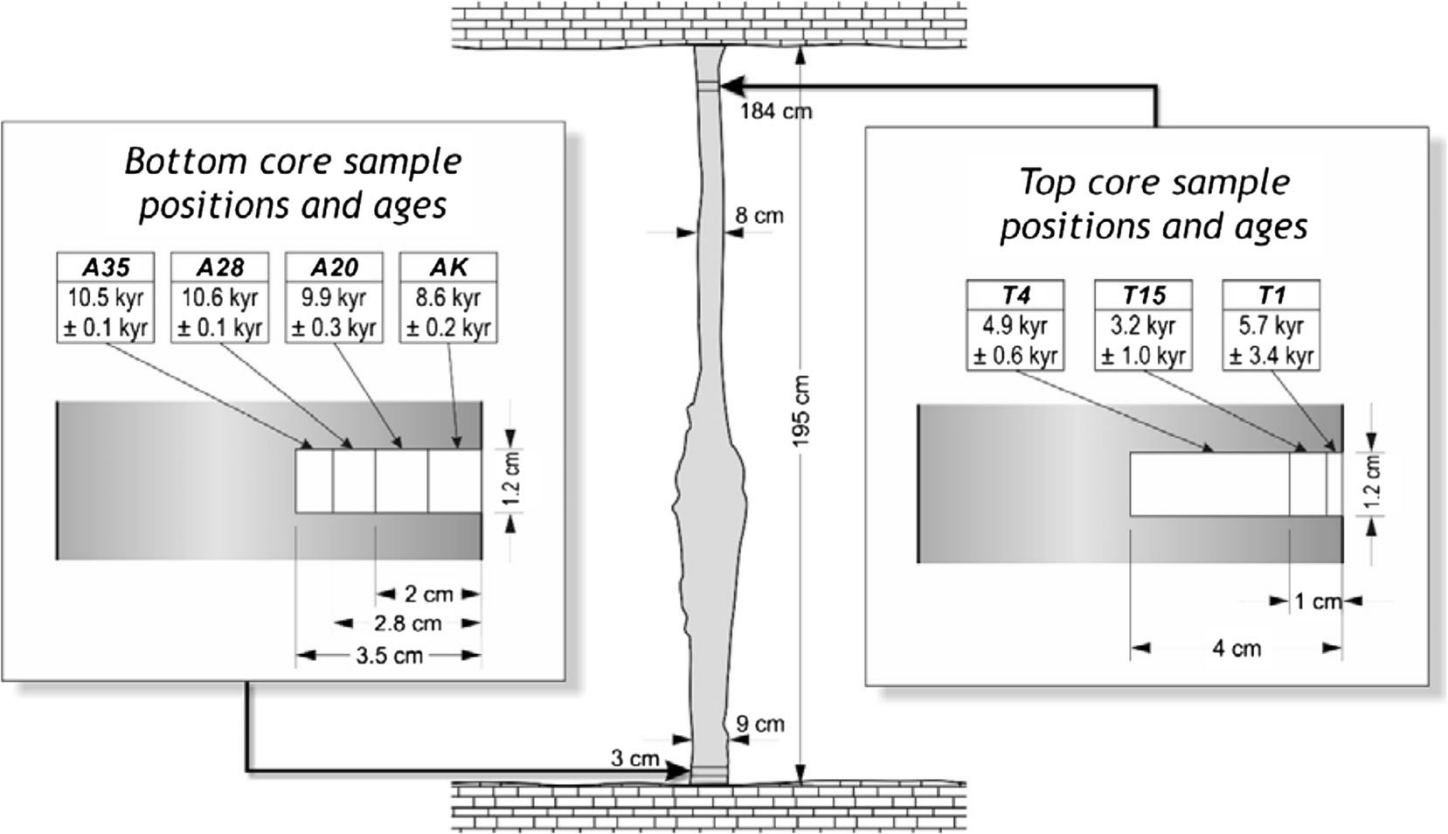
Location and age of cores drilled in the dripstone column for age dating

Because incremental sampling for age dating would likely have damaged this highly fragile stalagmite, therefore we could obtain core samples for age determination from only two places at the bottom and the middle part of the IVSTM (at the heights of 18 and 142 cm above the base, Fig. 9), and we did not take any core from the upper section of the stalagmite. We also obtained samples from a neighbouring 1.95-m-tall in situ dripstone column, which stands in the same chamber of the cave where the IVSTM is (Chamber of dripstones, Fig. 3). This neighbouring in situ dripstone column stands about 15 m from the location of the IVSTM. In this case, it was possible to drill core samples from the column at a higher position (184 cm) than in the IVSTM (top in Figs. 10 and 11). The reason is that columns are fixed to both the ceiling and to the ground making them statically stable formations (difficult to topple).

U and Th concentrations, isotopic ratios and age data (datum = 1950 AD) were measured at the University of Minnesota using multi-collector inductively coupled plasma mass spectrometry (MC-ICP-MS) (Thermo Fisher Neptune) using the protocols of Shen et al. (2012) and Cheng et al. (2013).

**Fig. 12:**
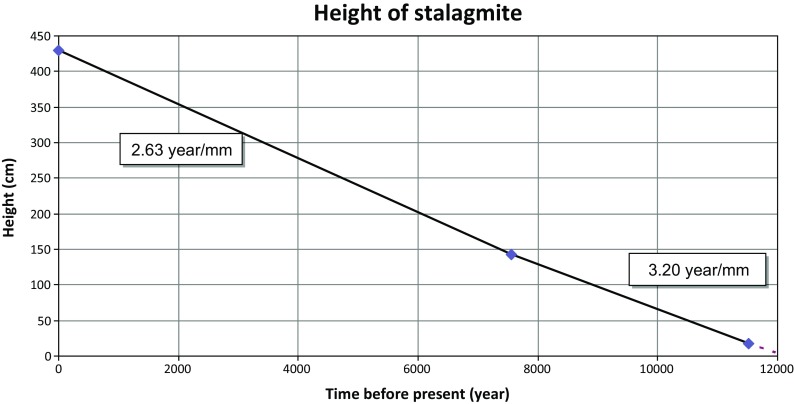
The height of the stalagmite we investigated as a function of time going back into the past calculated by the results of the age dating. The *numbers in the boxes* show the mean growth rate at two parts of the stalagmite

The results show that the column and the IVSTM formed during the same time interval at comparable growth rates (Table 6 and Figs. 11 and 12). The deposition of both speleothems began during the early Holocene about 10.5 and 11.4 ka (thousand years) ago. The top of the dripstone column (at 1.84 m) is about 5 ka old, and the middle part of the IVSTM (upper in Fig. 9, at 1.42 m) is about 7.5 ka old. The mean growth rate was about 3.0 year/mm (0.33 mm/year) in case of the column and 3.2 year/mm (0.31 mm/year) between 142 and 18 cm heights of the IVSTM, and it was about 2.6 year/mm (0.38 mm/year) between the peak of the IVSTM and 142-cm height (Fig. 12). The peak of the IVSTM is recent, because the IVSTM is always wet, i.e. it is still growing.

**Table 6:** ^230^Th dating results of age determination of core samples taken from the investigated stalagmite performed by MC-ICP-MS method

Height of sampling point (cm)	Cored interval (mm)	Sample number	^238^U (ppb)		^232^Th (ppt)		^230^Th/^232^Th (atomic ×10^−6^)		*δ* ^234^U^a^ (measured)		^230^Th/^238^U (activity)		^230^Th age (year) (uncorrected)		^230^Th age (year) (corrected)		*δ* ^234^U_Initial_^b^ (corrected)		^230^Th age (year BP)^c^ (corrected)	
142	21–24	GM 708	103.7	0.2	527	±11	275.0	6.1	265.8	±3.0	0.08474	±0.00077	7538	73	7422	110	271.5	3.1	7356	±110
142	30–31.5	GM 709	93.7	0.2	707	±14	185.7	4.9	255.7	±3.0	0.08499	±0.00142	7625	133	7451	181	261.1	3.1	7385	±181
142	24–30	GM 731	95.2	0.1	353	±7	380.1	9.8	265.5	±3.2	0.08543	±0.00132	7604	123	7519	137	271.2	3.2	7453	±137
142	31.5–34.5	GM 732	89.8	0.1	414	±8	307.3	7.6	261.9	±2.0	0.08588	±0.00119	7668	111	7562	134	267.5	2.1	7496	±134
18	0–2	GM 774	349.7	0.9	2221	±45	305.3	6.5	289.0	±3.0	0.11760	±0.00081	10,397	79	10,254	128	297.5	3.1	10,188	±128
18	2–6.5	GM 770	125.9	0.4	594	±12	423.4	9.7	277.0	±2.9	0.12117	±0.00132	10,833	127	10,726	147	285.5	3.0	10,660	±147
18	6.5–19	GM 771	73.8	0.2	882	±18	173.2	4.3	281.7	±3.6	0.12555	±0.00188	11,201	179	10,931	261	290.5	3.7	10,865	±261
18	19–27	GM 733	75.8	0.1	201	±4	771.5	18.5	274.3	±2.2	0.12406	±0.00135	11,130	128	11,070	135	283.0	2.3	11,004	±135
18	27–34	GM 734	68.3	0.1	578	±12	247.1	6.4	272.2	±3.4	0.12678	±0.00205	11,405	196	11,213	239	281.0	3.5	11,147	±239
18	34–39	GM 735	65.1	0.1	778	±16	177.7	4.0	259.6	±2.2	0.12893	±0.00133	11,733	129	11,458	233	268.2	2.3	11,392	±233
18	39–42	GM 736	58.9	0.1	158	±3	761.7	18.5	268.0	±2.2	0.12408	±0.00145	11,191	139	11,130	145	276.5	2.3	11,064	±145

The error is 2*σ* error. Corrected ^230^Th ages assume the initial ^230^Th/^232^Th atomic ratio of 4.4 ± 2.2 × 10^−6^. Those are the values for a material at secular equilibrium, with the bulk earth ^232^Th/^238^U value of 3.8. The errors are arbitrarily assumed to be 50%

^a^ *δ* ^234^U = ([^234^U/^238^U]_activity_ − 1) × 1000

^b^ *δ* ^234^U_initial_ was calculated based on ^230^Th age (*T*), i.e. *δ* ^234^U_initial_ = *δ* ^234^U_measured_ × *e* ^*λ*234 *× T*^

^c^BP stands for ‘before present’ where the ‘present’ is defined as the year 1950 AD

Similarly high growth rates were measured in Katerloch cave, in the Alps in Austria (0.2–0.7 mm/year), 180 km southwest of the study area (Boch et al. 2006, 2010). On the other hand, growth rates reported from stalagmites in Baradla and Domica caves, located 230 km east of the study area, are significantly lower (16 and 53 year/mm, respectively—Szeidovitz et al. 2008a; Gribovszki et al. 2013b).

## Changes in stalagmite shape over time

**Fig. 13:**
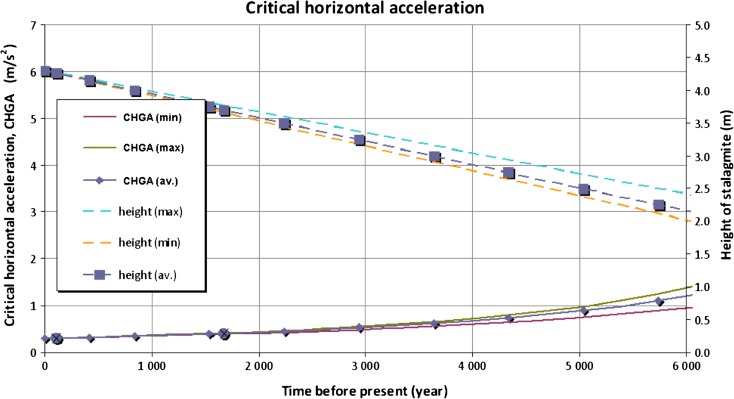
Heights of the IVSTM we investigated and the critical horizontal ground acceleration (CHGA) provided by its height as a function of time going back into the past as a function of the age of the stalagmite. The *curves marked by boxes* show the average values, and the *unmarked curves* show the minimum and maximum possible values (see text). The *two dots* on the CHGA curve belong to the Jókő and Carnuntum events

The results of the age determination suggest a simple model of the changing shape of the IVSTM we studied going back into the past (Fig. 13).

The stalagmite is still active (since it is wet), and we assume a constant growth rate, i.e. the average of the two determined growth rates (2.6 and 3.2 year/mm) of the IVSTM.

Candlestick-shaped stalagmites have almost cylindrical forms. Our assumption is that the candlestick-shaped stalagmites have almost cylindrical forms during their evaluation process as well underlie the papers of Dreybrodt and Romanov (2008) and Kaufmann (2003). These authors claimed that candlestick-shaped stalagmites grow with an assumed nearly constant diameter at their horizontal cross section.

The varying heights (and shapes) of the stalagmite going back in time as a linear function of the age of the stalagmite (Fig. 13 ‘height’ curves) were calculated, taking into account the previously mentioned assumptions (constant growth rate and constant diameter at their horizontal cross section). Knowing the height and diameter of the stalagmite at a given time in the past, the critical horizontal ground acceleration (CHGA) value was calculated corresponding to the height (and shape) of the stalagmite in the past (Figs. 13 ‘CHGA’ curves and 14 ‘in the cave’ curve) by the same method as before (Eq. (2)).

**Fig. 14:**
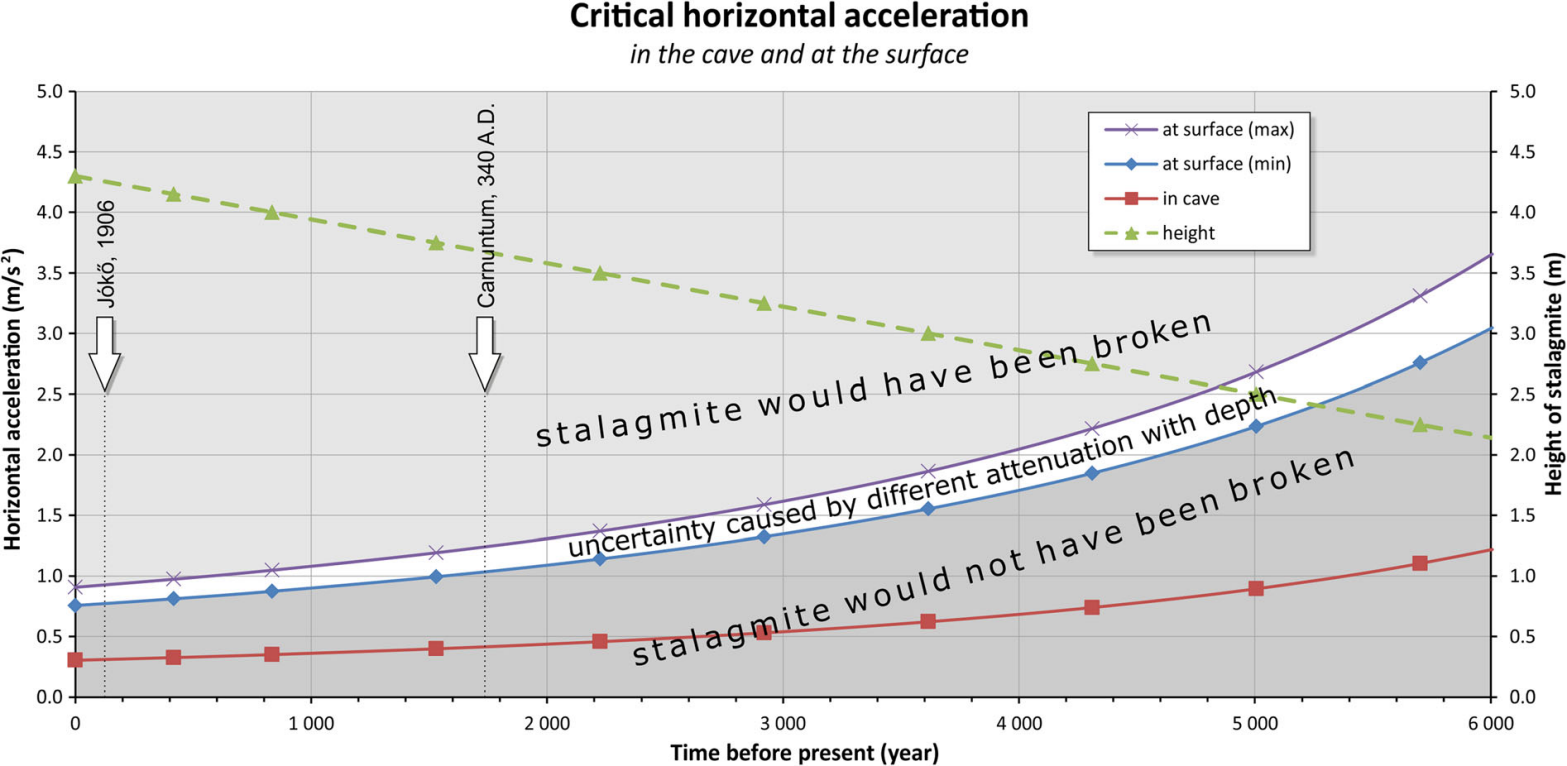
Constraints on critical horizontal ground acceleration (CHGA) at the surface and in the cave provided by the height of the IVSTM, which is still intact and provided by the depth of the cave. Ages of the assumed and real earthquakes in the area are also shown. The uncertainty of the CHGA at the surface is given by the *white region* (see text)

For example, the IVSTM was about 3.70 ± 0.08 m tall 1676 years ago, and the constant diameter of the stalagmite was 0.085 m. Note that Fig. 13 shows that the horizontal ground motion generated by the large earthquake, which may have destroyed the Roman legionary camp at Carnuntum (not prior to 340 AD), could not have been larger than 0.41 ± 0.02 m/s^2^. Otherwise, the stalagmite we investigated would have been broken. (The CHGA value for 340 AD is shown in Fig. 13.)

Figure 13 illustrates the uncertainties both in the height and CHGA values as well which result from the uncertainty in the growth rate (see the unmarked lines and curves). Since the growth rate of different parts of the IVSTM and the growth rate measured at the dripstone column agree (2.6, 3.2, 3.0 year/mm), therefore we can state that the used average growth rate cannot produce larger than 0.4 m/s^2^ bias at the CHGA results at a 6-ka time interval.

## Attenuation of seismic waves with depth

Since seismic waves are progressively attenuated with depth (Becker et al. 2006), it is important to know the depth of the cave, where the IVSTM we investigated is located. We also assume that the depth of the cave did not change considerably with time. Taking into account the thickness of the rock overburden, the chamber, where the stalagmite we investigated is located, it is situated at about 175 m below the surface, since the height difference between the cave entrance and the highest point of the hill is about 110 m.

Various studies have analysed how the acceleration of seismic waves changes with depth in mines and boreholes (Shimizu et al. 1996; Hu and Xie 2004; Iwasaki et al. 1977; Lednická and Kaláb 2016). Based on these measurements, we deduce that the PGA of seismic waves at 150–200-m depth should be attenuated by a factor of about 2.5–3 compared to the surface. Therefore, depending on the height of the stalagmite, the CHGA must be multiplied by 2.5 or 3 (more conservative assumption) in order to obtain the CHGA value at the surface. Figure 14 shows CHGA values going back in time in the cave and at the surface as well. If the horizontal ground acceleration values at the surface had reached or exceeded the values of ‘at the surface’ curve (or higher), then the stalagmite in the cave would have been broken (in the past). Since the stalagmite we investigated is still standing intact in the cave, therefore it cannot have happened.

Figure 14 shows as well how the propositions of prehistoric and real earthquakes can be tested using the IVSTM we investigated by providing long-term upper bounds on the horizontal ground acceleration.

## Discussion: the Jókő earthquake (Dobra Voda earthquake, 9 January 1906, 23:05 GMT)

In Sect. 3, we shortly mentioned the assumed moderate and large paleoearthquakes (occurred at the normal faults in the Vienna Basin) and prehistoric earthquakes (Carnuntum event, 340 AD) occurred at the wider surroundings of the Plavecká priepast cave (60–70 km). The effects of these earthquakes on the stalagmite we investigated would require a larger comprehensive study, which is far beyond the frame of the present paper. Here, we discuss only the effect of the Jókő earthquake (1906), since this is a most suitable event for our purposes (well documented in detail, larger event) at the near vicinity of the Plavecká priepast cave.

**Fig. 15:**
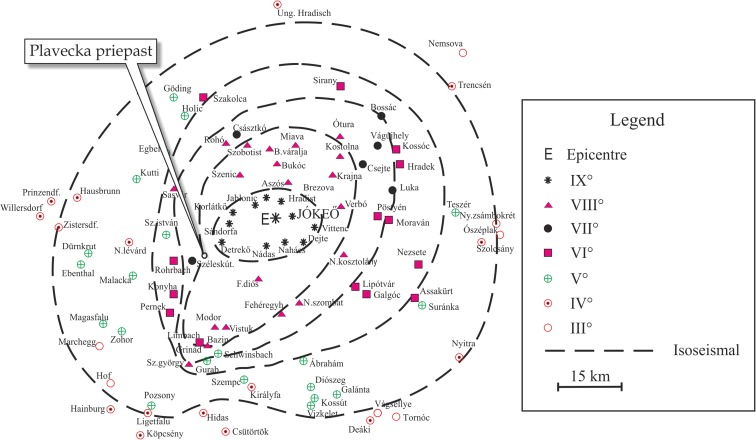
Central part of historical isoseismal map of the Jókő (Dobra Voda) earthquake which occurred on 10 January 1906, 00:05 (local time) on the Forel–Mercalli scale (Réthly 1907) and the location of the Plavecká priepast cave

On 10 January 1906, 00:05 (local time), an earthquake of *M* = 5.7 occurred about 20 km to the northeast of the Plavecká priepast cave. The epicentre was in Jókő (Dobra Voda). On 16 January 1906, i.e. 6 days later, another moderate earthquake of *M* = 5.3 occurred. The second earthquake was much less destructive than the first one. These two known historical events were the two largest ones close to the cave. Figure 15 shows the central part of the historical isoseismal map of the first earthquake (Réthly 1907). This map was prepared on the (historical) Forel–Mercalli scale.

The epicentral intensity was *I*_0_ = 9°, as determined on the Forel–Mercalli scale, after Réthly (1907). Zsíros (2005) re-estimated the effects of the earthquake on EMS-98 by using all the written information about the earthquake and calculated that it was *I*_0_ = 8°. Macroseismic estimations of the effect of the earthquake were registered in the surroundings of the Plavecká priepast cave in two villages: Detrekő and Széleskút (Fig. 15). Note that Detrekő is called Detrekőszentpéter, and Széleskút is called Pozsonyszéleskút in the Réthly Annual Report (Réthly 1907). According to Réthly (1907), the intensities felt in Detrekő and Széleskút were *I*_*n*_ = 9° and *I*_*n*_ = 7°, respectively, on the Forel–Mercalli scale.

Réthly (1907) reported that “The force of the earthquake decreased rapidly to the West, and it was rather difficult to draw the VIII° isoseismal curve between the IX° and the VII°, for the same reason.” The rapid decrease of the earthquake effect may be due to heterogeneous subsurface structure in the mountain chain or topographic scattering.

It is important to mention that the Little Carpathians are located between the hypocentre of the Jókő earthquake and the Plavecká priepast cave, which is situated southwest of the hypocentre.

The intensities on the EMS-98 scale, re-estimated by Zsíros (2005), were *I*_*n*_ = 7° for Detrekő and *I*_*n*_ = 5° for Széleskút. (Detrekőszentpéter is about 6.5 km northeast, and Pozsonyszéleskút is about 3 km southwest of the cave.)

Taking into account the result of Zsíros (2005) and the location of the cave between Detrekő and Széleskút, intensity 6° is more likely than intensity 7° on the EMS-98 scale at the location of the Plavecká priepast cave. This EMS-98 intensity (6°) is consistent with the results of our study of an IVSTM in the Plavecká priepast cave, because we state that at the time of the Jókő event (1906), the CHGA cannot have exceeded 0.31 m/s^2^ (in the cave). To calculate the CHGA values at the surface, this value has to be multiplied by 2.5 or 3, which results in 0.77 and 0.92 m/s^2^ values (Fig. 14). The peak ground motion is between 0.25 and 0.50 m/s^2^ according to Bisztricsány (1974) in case of intensity 6° on MSK scale. Grünthal et al. (1998) stated that “in most cases there should have been no difficulty in converting between MSK values and EMS values on the system MSK = EMS.” This means that the stalagmite we investigated cannot have been broken by the effect of the Jókő earthquake.

Studying the isoseismal map of the Jókő earthquake (Réthly 1907), we can recognize that the effect of the Jókő earthquake could be 6°–7° in EMS-98 scale at the location of today’s Bohunice Nuclear Power Plant, which is situated at the village of Jaslovské Bohunice (Apátszentmihály) between the villages of Horné Orešany (the former Felsődiós) and Velké Kostolany (the former Kosztolány) (Fig. 15). The nuclear powerplant is about 17 km from the epicentre. The effect of the Jókő earthquake on both villages was ∆ (=8°) on the Forel–Mercalli scale. The intensities on the EMS-98 scale, re-estimated by Zsíros (2005), were *I*_*n*_ = 6°–7° for both villages (Felsődiós and Kosztolány). This means that the destructive effect of the Jókő earthquake at this NPP could have been higher than at the Plavecká priepast cave.

## Conclusions

An IVSTM from the Plavecká priepast cave with a candlestick shape, tall, slim, cylindrical with a large height–diameter ratio, was examined to estimate the upper limit of horizontal peak ground acceleration generated by prehistoric earthquakes. Such IVSTMs have survived all earthquakes that have occurred over their long ‘lifetime’, often thousands of years. Their ‘survival’ requires that the horizontal ground acceleration has never exceeded a certain critical value within that period.

Our first results are restricted to a single cave, the Plavecká priepast, and a single IVSTM in the Little Carpathians of Slovakia.

The dimensions of the IVSTM were measured, and its vibration was recorded in situ. The natural frequency and the harmonic oscillations of the IVSTM were determined by analysing the recorded vibration.

The tensile failure stress, the density and the elastic properties of broken speleothem specimens were determined in geomechanical laboratory.

Based on a simple mechanical model, the theoretical natural frequency (*f*_0_), the harmonic oscillations (*f*_1_, *f*_2_) and the horizontal ground acceleration values resulting in a failure (*a*_*g*_) were calculated for the IVSTM using the results of the in situ investigations and the geomechanical laboratory measurements.

The measured natural frequency of the IVSTM (3 Hz) is almost the same as the calculated one (2.3 Hz). The theoretical harmonic oscillations (*f*_1_, *f*_2_) were calculated by two different methods. The theoretical values fit well to the measured ones. The theoretical-measured approach harmonic oscillation values are slightly higher than the measured ones, which means that the shape of this stalagmite is not completely cylindrical, but the diameters of the cross sections decrease upward along the vertical axis of the stalagmite we investigated.

Based on laboratory measurements and simple mechanical calculations for the static case, we found that at the present time, the horizontal ground acceleration resulting in a failure of the IVSTM in the Plavecká priepast cave is *a*_*g*_ = 0.30 m/s^2^ in the cave and 0.91 m/s^2^ at the surface. Even a small or moderate size earthquake can cause this ground acceleration value. Given that the modelling of the IVSTM in the Plavecká priepast cave was remarkably successful with regard to the *f*_0_ value, the resulting acceleration value *a*_*g*_ = 0.30 m/s^2^ can be considered reliable, for the static case.

The age and the growth rate of a dripstone column from the Plavecká priepast cave and of the IVSTM were determined by taking core samples at two different heights. The results show that this dripstone column and the IVSTM are from the Holocene epoch, and the mean growth rate is between 2.6 and 3.2 year/mm, i.e. quite high. Although the age data are sparse, the extremely good fit with a constant growth rate curve suggests an uninterrupted evolution of the stalagmite.

The stalagmite we investigated from the Plavecká priepast cave provides CHGA values at different times going back into the past. If one takes into account the attenuation of seismic waves with distance (GMPEs), then the IVSTM can indicate whether or not large prehistoric earthquakes occurred in the surroundings of the Plavecká priepast cave (e.g. assumed Carnuntum event occurred not earlier than 340 AD).

This technique can yield important constraints on the seismic hazard in this region, because geological structures close to the cave apparently did not generate strong paleoearthquakes in the last few thousand years, which would have produced horizontal ground acceleration larger than the upper acceleration threshold determined from the IVSTM.

We compared the effect of the Jókő (Dobra Voda) earthquake (10 January 1906) to the upper limit of the horizontal ground motion calculated from the IVSTM we investigated. We estimated that the effect of the Jókő earthquake was about 6° on the EMS-98 intensity scale at the location of the cave based on the macroseismic isoseismal curves of the event. This intensity is consistent with the result of this study.

Even if the new constraints provide information only from a single point in space, the constraints are very valuable since it is complementary to earthquake catalogues, which are limited to much shorter time intervals. These new results are relevant when assessing the seismic potential of faults close to the Plavecká priepast cave (Mur–Mürz–Vienna Basin–Zilina line), as well as of the VBTF system. The seismic hazard of the two nearby capitals Vienna and Bratislava is particularly important.

The appearance and frequency of large destructive events in the VBTF system raise an open question. To resolve this, it is necessary to know the attenuation of seismic waves with distance (GMPE). This requires detailed inspection because the isoseismal contours of the historical macroseismic maps in the area have a strongly asymmetric shape. This is the reason why a comprehensive study is necessary to assess the existence of a seismic gap in VBTF system. This kind of study is beyond the scope of this paper: A detailed investigation about this wider topic has already been started. More precise conclusions on seismic hazard of the two nearby capitals Vienna and Bratislava will require the measurement of seismic wave attenuation with distance in the area. This is feasible, and it will be done in the future.

## References

[CR1] Anderson JG, Brune JN, Biasi G, Anooshehpoor A, Purvance M (2011). Workshop report: applications of precarious rocks and related fragile geological features to U.S. national hazard maps. Seismol Res Lett.

[CR2] Becker A, Davenport CA, Eichenberger U, Gilli E, Jeannin P-Y, Lacave C (2006). Speleoseismology: a critical perspective. J Seismol.

[CR3] Bednárik M (2009) Seismometric portrayal of calcite tubular stalactites. Ph.D. thesis. Geophysical Institute, Slovak Academy of Sciences, p. 146

[CR4] Beidinger A, Decker K (2011). 3D geometry and kinematics of the Lassee flower structure: implications for segmentation and seismotectonics of the Vienna Basin strike–slip fault. Austria Tectonophysics.

[CR5] Beyreuther M, Barsch R, Krischer L, Megies T, Behr Y, Wassermann J (2010). ObsPy: apython toolbox for seismology. SRL.

[CR6] Bisztricsány E (1974) Mérnökszeizmológia. (Engineering Seismology, in Hungarian), Akadémiai Kiadó, Budapest, 216p

[CR7] Boch R, Spötl C, Kramers J (2006) Das Alter der Stalagmiten im Katerloch (2833/59): Erste Ergebnisse der Uran/Thorium Datierung, Die Höhle / 57. Jg. / Heft 1-4

[CR8] Boch R, Spötl C, Kramers J (2010) Wachstumsphasen von Stalagmiten im Katerloch (2833/59), Die Höhle / 61. Jg / Heft 1-4

[CR9] Budó Á (1968). Kísérleti fizika I. (experimental physics I., in Hungarian).

[CR10] Brune JN, Whitney JW (2000) Precarious rocks and seismic shaking at Yucca Mountain, Nevada, In: Whitney, JW, Keefer WR (eds.), Geologic and Geophysical Characterization Studies of Yucca Mountain, Nevada, A Potential High-Level Radioactive-Waste Repository, U.S. Geological Survey Digital Data Series 58, Chapter M

[CR11] Butáš J (2003). Plavecká priepasť PP-2. Spravodaj Slovenskej speleologickej spoločnosti (Bulletin of Slovak Speleological Society).

[CR12] Butáš J(2005) Plavecká Abyss PP-2. Bulletin of Slovak Speleological Society, special edition, Proceedings of 14th Speleological Congress UIS in Greece, 48–50

[CR13] Cadorin J, Jongmans D, Plumier A, Camelbeeck T, Delaby S, Quinif Y (2001). Modelling of speleothems failure in the Hotton cave (Belgium). Is the failure earthquake induced?. Neth J Geosci.

[CR14] Cheng H, Edwards RL, Shen C-C, Polyak VJ, Asmerom Y, Woodhead J, Hellstrom J, Wang YJ, Kong XG, Spötl C, Wang XF, Alexander EC (2013). Improvements in Th-230 dating, Th-230 and U-234 half-life values, and U-Th isotopic measurements by multi-collector inductively coupled plasma mass spectrometry. Earth Planet Sci Lett.

[CR15] Csák B, Hunyadi F, Vértes Gy (1981) Földrengések hatása az éptményekre (Effect of earthquakes upon structures, in Hungarian), Műszaki könyvkiadó, Budapest, p. 355

[CR16] Decker K, Gangl G, Kandler M (2006). The earthquake of Carnuntum in the fourth century A.D.*—*archaeological results, seismologic scenario and seismotectonic implications for the Vienna Basin fault, Austria, J. Seismology.

[CR17] Dreybrodt W, Romanov D (2008). Regular stalagmites: theory behind their shape. Acta Carsologica.

[CR18] Fairchild J, Baker A (2012) Speleothem science: from process to past environments. Wiley-Blackwell, p. 416

[CR19] Forti P(1997) Speleothems and earthquakes. Cave Minerals of the World, Second edition, National Speleological Society, USA, 284–285

[CR20] Forti P(1998) Seismotectonic and paleoseismic studies from speleothems: the state of the art. HAN 98, Sp’el’eochronos Hors-S’erie, 79–81

[CR21] Forti P (2001). Biogenic speleothems: an overview. Int J Speleol.

[CR22] Gribovszki K, Paskaleva I, Kostov K, Varga P, Nikolov G (2008) Estimating an upper limit on prehistoric peak ground acceleration using the parameters of intact speleothems in caves in southwestern Bulgaria. 287–308, In: Zaicenco A, Craifaleanu I, Paskaleva I (eds) Harmonization of Seismic Hazard in Vrancea Zone with Special Emphasis on Seismic risk Reduction (NATO Science for Peace and Security, Series C: Environmental Security) Dordrecht: Springer Verlag, p. 347 (ISBN: 978–1–4020-9241-1)

[CR23] Gribovszki K, Bokelmann G, Szeidovitz GY, Varga P, Paskaleva I, Brimich L, Kovács K (2013a) Comprehensive investigation of intact, vulnerable stalagmites to estimate an upper limit on prehistoric ground acceleration. Proceedings of the Vienna Congress on Recent Advanced in Earthquake Engineering and Structural Dynamics & 13. D-A-CH Tagung, Vienna, No. 445

[CR24] Gribovszki K, Kovács K, Mónus P, Shen C-C, Török Á, Brimich L (2013). Estimation of an upper limit on prehistoric peak ground acceleration using the parameters of intact stalagmites and the mechanical properties of broken stalagmites in Domica cave, Slovakia. Slovensky kras. Acta Carsologica Slovaca.

[CR25] Grünthal G (ed.) (1998) European Macroseismic Scale 1998 (EMS-98). Cahiers du Centre Européen de Géodynamique et de Séismologie 15, Centre Européen de Géodynamique et de Séismologie, Luxembourg, p. 99

[CR26] Hammerl C, Loecker K, Steffelbauer I, Totschnig R (2014) The Carnuntum case—an earthquake catastrophe around 350 A.D.? EGU general assembly. Geophysical Research Abstracts 16:EGU2014–EG12678

[CR27] Hintersberger E, Decker K, Lomax J, Fiebig M, Lüthgens C (2013) Fault linkage model of strike-slip and normal faults in the Vienna Basin based on paleoseismological constraints. EGU General Assembly, Geophysical Research Abstracts 15:EGU2013–12755

[CR28] Hintersberger E, Decker K (2014). A seismic gap at the Central Vienna Basin Transfer Fault (Vienna Basin, Austria)?. EGU General Assembly, Geophysical Research Abstracts.

[CR29] Horváth F, Bada G, Windhoffer G, Csontos L, Dövényi P, Fodor L, Grenerczy GY, Sikhegyi F, Szafián P, Székely B, Timár G, Tóth L, Tóth T (2004) Atlas of the present-day geodynamics of the Pannonian basin: Euroconform maps with explanatory text

[CR30] Hu J, Xie L (2004). Variation of earthquake ground motion with depth. Acta Seismol Sin.

[CR31] Iwasaki T, Wakabayashi S, Tatsuoka F (1977) Characteristics of underground seismic motions at four sites around Tokyo Bay. In: Lew, HS (ed.), Wind and seismic effects, Proceedings of the Eighth Joint Panel Conference of the U. S. – Japan Cooperative Program in Natural Resources, Nat. Bur. Stand. (U.S.), Spec. Publ. 477, U.S. Government Printing Office, Washington, III-41 – III-56.

[CR32] Kagan EJ, Agnon A, Bar-Matthews M, Ayalon A (2005). Dating large infrequent earthquakes by damaged cave deposits. Geology.

[CR33] Kaufmann G (2003). Stalagmite growth and palaeo-climate: the numerical perspective. EPSL.

[CR34] Konecny P, Lednická M, Soucek K, Stas L, Kubina L, Gribovszki K (2015). Determination of dynamic Young’s modulus of vulnerable speleothems. Acta Montan Slovaca.

[CR35] Kong S, Zhou S, Nie Z, Wang K (2008). The size-dependent natural frequency of Bernoulli-Euler micro-beams. Int J Eng Sci.

[CR36] Labák P, Bystrická A, Moczo P, Campbell KW, Rosenberg L(1998) Preliminary probabilistic seismic hazard assessment for the Nuclear Power Plant Bohunice (Slovakia) site. In: Bisch, P. et al., Proc. of the 11th ECEE, Balkema, Rotterdam (12p, CD-ROM)

[CR37] Lacave C, Levret A, Koller M, (2000) Measurements of natural frequencies and damping of speleothems. Proc. of the 12^th^ World Conference on Earthquake Engineering, Auckland, New-Zealand, No. 2118.

[CR38] Lacave C, Koller MG, Egozcue JJ (2004). What can be concluded about seismic history from broken and unbroken speleothems?. J Earthq Eng.

[CR39] Lednická M, Zdeněk K (2016). Vibration effect of near earthquakes at different depths in a shallow medieval mine. Acta Geophysica.

[CR40] Nováková L, Sosna K, Broz M, Najser J, Novak P (2011). Geomechanical parameters of the podlesi granites and their relationship to seismic velocities. Acta Geodyn Geomater.

[CR41] Mahel’, M, (ed.) (1972) Geological Map of Malé Karpaty Mts., M = 1:50000, GUDŠ Bratislava

[CR42] Paskaleva I, Szeidovitz G, Kostov K, Koleva G, Nikolov G, Gribovszki K, Czifra T (2006). Calculating the peak ground horizontal acceleration generated by paleoearthquakes from failure tensile stress of speleothems.

[CR43] Paskaleva I, Gribovszki K, Kostov K, Varga P, Nikolov G (2008). Peak ground acceleration assessment using the parameters of intact speleothems in caves situated in NW and SW Bulgaria.

[CR44] Panno SV, Craig CL, Hackley KC, Curry BB, Fouke BW, Zhang Z (2009). Major earthquakes recorded by speleothems in midwestern U.S. caves. Bull Seism Soc Am.

[CR45] Réthly A (1907) Az 1906. évi magyarországi földrengések (Earthquakes occurred in Hungary in 1906, in Hungarian), Országos Meteorológiai és Földmágnességi Intézet, Budapest, p. 204

[CR46] Scholz H (1990) The mechanics of the earthquakes and faulting. Cambridge University Press, p. 467 10.1126/science.250.4988.1758-a 17734719

[CR47] Šebela S (2008). Broken speleothems as indicators of tectonic movements. Acta Carsologica.

[CR48] Shanov S, Kostov K (2015) Dynamic tectonics and karst, cave and karst systems of the world. Springer-Verlag Berlin Heidelberg, p. 123

[CR49] Shen C-C, Wu C-C, Cheng H, Edwards RL, Hsieh Y-T, Gallet S, Chang C-C, Li T-Y, Lam DD, Kano A, Hori M, Spötl C (2012). High-precision and high-resolution carbonate 230Th dating by MC-ICP-MS with SEM protocols. Geochim Cosmochim Acta.

[CR50] Shimizu I, Osawa H, Seo T, Yasuike S, Sasaki S (1996). Earthquake-related ground motion and groundwater pressure change at the Kamaishi Mine. Eng Geol.

[CR51] Šmída, B (2010) Geomorfológia a genéza Plaveckého krasu ako modelového územia tzv. kontaktného krasu Západných Karpát s nižšou energiou reliéfotvorby. (Geomorphology and formation of Detrekő karst) Dizertačná práca, Katedra fyzickej geografie a geoekológie, Prírodovedecká fakulta Univerzity Komenského v Bratislave, p. 220 (in Slovak)

[CR52] Szeidovitz G, Leél-O”ssy S, Surányi G, Czifra T, Gribovszki K (2005). Calculating the peak ground horizontal acceleration generated by paleoearthquakes from failure tensile stress of speleothems. Hungarian Geophysics.

[CR53] Szeidovitz G, Paskaleva I, Gribovszki K, Kostov K, Surányi G, Varga Z, Nikolov G (2008). Estimation of an upper limit on prehistoric peak ground acceleration using the parameters of intact speleothems in caves situated at the western part of Balkan Mountain Range. Acta Geod Geoph Hung.

[CR54] Szeidovitz G, Surányi G, Gribovszki K, Bus Z, Leél-O”ssy S, Varga Z (2008). Estimation of an upper limit on prehistoric peak ground acceleration using the parameters of intact speleothems in Hungarian caves. J Seismol.

[CR55] Tóth L, Mónus P, Zsíros T, Bondár I, Bus Z, Kosztyu Z, Kiszely M, Wéber Z, Czifra T (1996-2014) Hungarian earthquake bulletin, 1995–2013. MTA GGKI és Georisk Kft, Budapest

[CR56] Wessel P, Smith WHF (1998). New improved version of generic mapping tools released. Eos.

[CR57] Zsíros T (2000) The seismicity and earthquake hazard of the Pannonian basin: Hungarian earthquake catalog (456–1995, in Hungarian). MTA GGKI Szeizmológiai Obszervatórium, p. 482

[CR58] Zsíros T (2005). Seismicity of the Western-Carpathians. ACTA Geod Geophys Hung.

